# Nitric oxide production in murine spleen cells: role of interferons and prostaglandin E_2_ in the generation of cytotoxic activity

**DOI:** 10.1155/S0962935196000117

**Published:** 1996-02

**Authors:** Dominique Vaillier, Richard Daculsi, Norbert Gualdel

**Affiliations:** 1 URA 1456 CNRS Université de Bordeaux II France; 2 Institut Bergonié Bordeaux France

## Abstract

The production of nitric oxide (NO) was measured in cultures of spleen cells stimulated by lipopolysaccharide (LPS), IL-2 or LPS + IL-2. We observed that NO synthesis is increased by IFN-γ but inhibited by IFN-α/β. This is not the case when IL-2 is present in the cultures, since interferons play a minor role in the regulation of the NO production. When IL-2 and LPS were associated in the cultures, the IFN-α/β role seems more important than that of IFN-γ. PGE_2_ inhibits NO production in LPS supplemented cultures but has a slight effect in the presence of IL-2 and no effect with IL-2 + LPS. 3-isoButyl-1-methylxanthine (IBMX), an inhibitor of phosphodiesterases, induces a decrease of IFN production. In the presence of H-7, an inhibitor of protein kinase C (PKC), NO production is reduced when the cultures are supplemented by LPS or IL-2 but not when IL-2 and LPS are both added. H-7 also reduced IFN production. In the presence of N^G^-monomethyl-L-arginine (N-MMA), an inhibitor of NO synthesis, IFN production was increased, with no change in the cytotoxic activity. Hence, interferons regulate NO production by mouse spleen cells and, in return, NO modulates the generation of IFN.


Mediators of Inflammation 5, 62–68 (1996)

### Nitric oxide production in murine spleen cells: role of interferons and prostaglandin $E_2$ in the generation of cytotoxic activity

Dominique Vaillier,[1] Richard Daculsi[1,CA] and Norbert Gualde[1,2]

[1]URA 1456 CNRS, Université de Bordeaux II, France. Fax: (+33) 56 333330; [2]Institut Bergonié, Bordeaux, France.

[CA]Corresponding Author

**The production of nitric oxide (NO) was measured in cultures of spleen cells stimulated by lipopolysaccharide (LPS), IL-2 or LPS + IL-2. We observed that NO synthesis is increased by IFN-γ but inhibited by IFN-α/β. This is not the case when IL-2 is present in the cultures, since interferons play a minor role in the regulation of the NO production. When IL-2 and LPS were associated in the cultures, the IFN-α/β role seems more important than that of IFN-γ. $PGE_2$ inhibits NO production in LPS supplemented cultures but has a slight effect in the presence of IL-2 and no effect with IL-2 + LPS. 3-*iso*Butyl-1-methylxanthine (IBMX), an inhibitor of phosphodiesterases, induces a decrease of IFN production. In the presence of H-7, an inhibitor of protein kinase C (PKC), NO production is reduced when the cultures are supplemented by LPS or IL-2 but not when IL-2 and LPS are both added. H-7 also reduced IFN production. In the presence of $N^G$-monomethyl-L-arginine (*N*-MMA), an inhibitor of NO synthesis, IFN production was increased, with no change in the cytotoxic activity. Hence, interferons regulate NO production by mouse spleen cells and, in return, NO modulates the generation of IFN.**

**Key words:** Cytotoxicity, Interleukin-2, Lipopolysaccharide, Nitric oxide

### Introduction

Nitric oxide (NO) is important in many biological functions.[1] It is generated from L-arginine by the enzyme NO synthase (NOS). Peritoneal macrophages of mice are the best-characterized source of NO.

LPS and interferon-γ (IFN-γ) constitute the major stimulating factors of NO production[2] by increasing cellular concentrations of NOS.[3] Many functions of LPS have been reported to be mediated by prostaglandin production, which in turn acts by increasing cAMP.[4,5] The role of $PGE_2$ on NO production is unclear. $PGE_2$ did not affect NO production[6] nor did it inhibit NO production.[7] Other work shows that NO activates cyclooxygenase enzymes and leads to a marked increase in $PGE_2$ production.[8]

Intracellular mechanisms induced by LPS implicate protein kinase C (PKC) and cAMP dependent protein kinase A. LPS causes the translocation of protein kinase C from cytosol to the membrane.[9] PKC has been involved in the induction of NO synthase activity in macrophages by IFN-γ.[10] Another report suggested rather the role of protein kinase A.[11]

The studies on NO production and NO regulation have been performed essentially with purified macrophage populations. We have shown in previous work that LAK activity induced by IL-2 was reduced by LPS and that the decrease was related to the increase of IFN-α/β production.[12] On the other hand, NO was implicated in LAK activity in rodents.[13] Thus the aim of this study was to evaluate NO production induced by splenic macrophage cultures with other splenic cell populations and their cytokines after stimulation by LPS and IL-2, and to assess if there is a relationship between the generation of cytotoxic activity and NO production.

We have investigated whether NO, measured as nitrite ($NO_2^-$), is detected in supernatants of spleen cells stimulated for 3 days in the presence of LPS, IL-2 or LPS + IL-2, and the role played by interferons induced by IL-2 and LPS on NO generation. We have also examined whether NO production was regulated by $PGE_2$ and by LPS-induced PKC activation. Finally we have investigated whether NO plays a role in the generation of cytotoxic activity.

### Materials and Methods

*Mice:* Inbred C3H mice, 6–8 weeks old, were used throughout the experiments.

© 1996 Rapid Science Publishers

*Reagents:* Lipopolysaccharide from *Escherichia coli* 055:B5 was purchased from Difco. Anti-IFN-α/β serum (1:320 000) was a generous gift from Dr Ion Gresser (IRCS, Villejuif, France). Anti-murine IFN-γ antibodies were purchased from Genzyme (Boston, MA). rIL-2 was purchased from Cetus Corporation (Emeryville, CA) and $PGE_2$, IBMX, *N*-MMA and H-7 from Sigma (St Louis, MO).

*Cell lines:* YAC-1 and L929 cells were maintained *in vitro.* YAC-1 is a Moloney virus-induced lymphoma of A/Sn origin. L929 is a fibroblast cell line derived from an adult C3H mouse.

*Preparation of spleen cells:* Spleen cells were harvested and suspended in phosphate-buffered saline solution (PBS). Erythrocytes were removed by osmotic shock and the final cell suspension was resuspended in RPMI 1640 culture medium supplemented with 5% foetal calf serum, L-glutamine (2 mM), Hepes (10 mM), penicillin (100 U/ml), streptomycin sulfate (100 μg/ml), and 2-mercaptoethanol (0.05 mM), which constitutes complete medium.

*Measurement of $NO_2^-$ production:* NO was estimated by measuring the formation of $NO_2^-$ (the stable oxidative end product of NO) which serves as a quantitative index of macrophage activation. $NO_2^-$ levels in culture fluids were estimated by using the Griess reagent.[14] Briefly, 100 μl culture fluid was incubated with an equal volume of 1% sulfanilamide and 1% *N*-1-naphthylethylenediamine dihydrochloride in 2.5% $H_3PO_4$ (Sigma) at room temperature for 5 min. $NO_2^-$ was quantified by using spectrophotometry to measure the OD at 570 nm, with $NaNO_2$ as a standard.

*Inhibition of $NO_2^-$ synthesis:* The specific inhibitor of NO synthase, $N^G$-monomethyl-L-arginine (*N*-NMA) was added at a concentration of 50 μM to the spleen cell culture.

*Cytotoxicity assay:* Effector spleen cells were assayed using $^{51}Cr$-$Na_2CrO_4$-labelled (Amersham, UK) target cells. Cells from the YAC line were used as targets. Cytotoxic activity was measured for different spleen/target cell ratios in a final volume of 200 μl. After 4 h incubation at 37°C, 100 μl supernatants were removed from each well for counting. Specific lysis was calculated as:

$$\%\ \text{lysis} = \frac{\text{experimental release} - \text{spontaneous release}}{\text{total release} - \text{spontaneous release}} \times 100$$

Spontaneous release was obtained by incubating targets alone and total release was determined by collecting 100 μl from cells in which the pellet had been resuspended. All assays were performed in triplicate. The S.E.M. of the mean was within 5–10% and was not represented. Results of cytotoxicity assays were expressed either in percent of lysis for different effector/target cell ratios or in lytic units (LU)/$10^7$ cells. LU was calculated from linear regression curves plotted from the various E/T ratios. One LU was defined as the number of effector cells required to lyse 20% of the target cells.

*IFN assay:* The presence of IFN was assayed by anti-vesicular stomatitis virus (VSV) activity. L929 cells were seeded in 96-well ($3 \times 10^4$/well) flat-microtitre plates. Serial dilutions of supernatants were added. After 18 h at 37°C, the cells were infected with VSV at high multiplicity of infection. The plates were scored after 24 h. The antiviral effect was estimated by measuring the incorporation of red neutral dye into living cells according to the method described by Hudson and Hay[15] and using a Titertek multiscan spectrophotometer. The IFN titre of a supernatant was estimated as the reciprocal of the dilution inhibiting 50% of the cytopathic effect.

### Results

*Release of NO in spleen cells cultured in the presence of LPS, IL-2 or LPS + IL-2:* Initially we tried to determine whether under our experimental conditions, splenocytes could be stimulated by IL-2, LPS or both, to produce NO. For that purpose spleen cells were cultured for 3 days in the presence of increasing concentrations of the inducers used either alone or associated. $NO_2^-$ was assessed in supernatants by the Griess procedure. The results of one out of three similar experiments are shown in Fig. 1. We observed that the response to LPS was induced with low concentrations of reagent (1 ng/ml), increased and reached a plateau for a range of concentrations from 100 to 10 000 ng/ml. NO induction was possible with 100 U/ml of IL-2, and was even greater when LPS and IL-2 were present in the culture. One should underline that even if IL-2 alone at 10 U/ml did not induce NO synthesis it can act synergistically with LPS.

In a previous report[12] it was demonstrated that IL-2-induced LAK activity was diminished by LPS (1 μg/ml). As the aim of this work was to investigate whether NO production was involved in that decrease, the following experiments were performed with 1000 U/ml of IL-2 and 1 μg/ml of LPS.

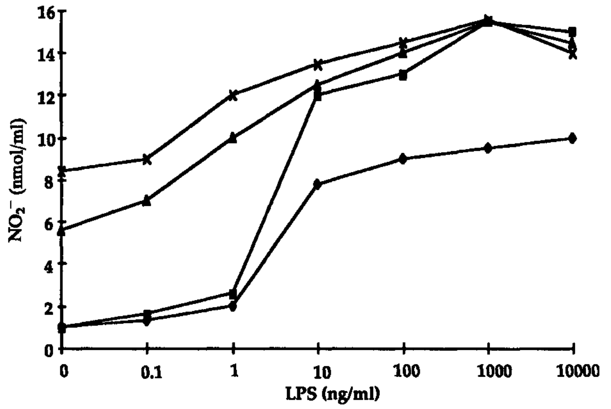

FIG. 1. Production of $NO_2^-$ in supernatant of spleen cells cultured for 72 h with various concentrations of LPS, IL-2 or with LPS + IL-2. The data are expressed as the mean from one representative experiment performed in triplicate (S.E.M. < 10%). ◆, Medium; ■, IL-2, 10 U/ml; ▲, IL-2, 100 U/ml; ×, IL-2, 1000 U/ml.

**Table 2.** NO variation (in percent) induced in presence of anti-IFN-γ or IFN-α/β antibodies in murine spleen cell cultured with LPS, IL-2 or LPS + IL-2

| Experiment | Substance(s) added | NO variation (%) | |
|---|---|---|---|
| | | anti-IFN-γ | anti-IFN-α/β |
| 1 | LPS | − 50[a] | +62 |
| | IL-2 | − 9 | +11 |
| | LPS + IL-2 | − 13 | +39 |
| 2 | LPS | − 78 | +48 |
| | IL-2 | 0 | 0 |
| | LPS + IL-2 | − 26 | +38 |
| 3 | LPS | − 43 | +30 |
| | IL-2 | 0 | +10 |
| | LPS + IL-2 | − 15 | +39 |

[a]Results were presented as the percentage of variation (enhancement or inhibition) induced by anti-IFN-γ or anti-IFN-α/β presence and calculated as A − B/B × 100, in which A is NO measured in the anti-IFN cultures and B is NO measured in cultures without anti-IFN.

*Implication of interferons in the production of nitric oxide:* IL-2 is an inducer of IFN-γ.[16] LPS is also an inducer of IFN-α/β[17] and the association of IL-2 with LPS induces a synergistic increase in the production of IFN-α/β and IFN-γ.[12,18] Moreover, IFN-α/β is implicated in the change in cytotoxic activity induced by LPS on IL-2-stimulated spleen cells.[12]

We have studied the effects of IFN-γ or IFN-α/β on NO production induced by LPS after 72 h of culture. For this purpose 100 U/ml of IFN-γ or IFN-α/β were added to spleen cells cultured in the presence of LPS at 1 μg/ml. Table 1 summarizes four experiments. It was observed that IFN-γ induced an increase of $NO_2^-$ (+ 128%) while IFN-α/β reduced $NO_2^-$ production (− 34%).

We then determined if the interferons induced by LPS, IL-2 or a mixture of both played a role in NO production. For that, the culture was incubated in the presence of anti-IFN-γ antibodies or anti-IFN-α/β anti-serum, and $NO_2^-$ was measured after 3 days. Table 2 presents the results of three experiments. We observed that for LPS cultures the anti-IFN-γ antibodies reduced NO production. This effect of anti-IFN-γ antibodies was diminished in cultures stimulated by IL-2 or IL-2 + LPS. The presence of anti-IFN-α/β serum increased NO production by 40% for LPS and IL-2 + LPS cultures, with smaller increases in IL-2 cultures.

*The role of cAMP on NO production by spleen cells cultured in the presence of IL-2 or IL-2 + LPS, in relation to IFN production:* LPS-stimulated macrophages produce $PGE_2$ which induces a transient increase of cAMP production.[6] cAMP is degraded by phosphodiesterases. For testing the effects of endogenous $PGE_2$ stimulated by LPS, we added IBMX, an inhibitor of phosphodiesterase, to the cultures and therefore promoted the accumulation of cAMP in the culture medium. NO production was measured after 3 days. Results were presented as the percent variation of NO between the IBMX group and the untreated group. The percent variation of five experiments is shown in Table 3. The produc-

**Table 1.** Effects of IFN-α/β or IFN-γ on $NO_2^-$ production by spleen cells cultured 72 h with LPS

| Experiment | Concentration of $NO_2^-$ (nmol/ml) | | |
|---|---|---|---|
| | LPS | LPS + IFN-γ[a] | LPS + IFN-α/β[a] |
| 1 | 8 | 31 | 5 |
| 2 | 10 | 22 | 6 |
| 3 | 15 | 30 | 10 |
| 4 | 17 | 31 | 12 |
| Mean ± S.E.M. | 12.5 ± 2.1 | 28.5 ± 2.0[b] | 8.2 ± 1.9[c] |

[a]Spleen cells were cultured for 72 h with LPS (1 μg/ml) added or not, with IFN-γ or IFN-α/β at 100 U/ml.

[b]$p < 0.01$ *vs.* LPS.

[c]$p < 0.05$ *vs.* LPS.

**Table 3.** Effect of a phosphodiesterases inhibitor (IBMX) on the NO produced by LPS, IL-2 or LPS + IL-2 stimulated spleen cells cultured for 3 days

| Experiment | NO variation (%)[a] | | |
|---|---|---|---|
| | LPS | IL-2 | LPS + IL-2 |
| 1 | − 73 | − 32 | − 21 |
| 2 | − 80 | +23 | − 36 |
| 3 | − 58 | − 50 | − 9 |
| 4 | − 74 | − 33 | − 39 |
| 5 | − 92 | − 10 | +25 |
| Mean ± S.E.M. | − 75.4 ± 5.5 | − 20.4 ± 12.6 | − 16.2 ± 11 |

[a]% variation = A − B/B × 100 in which A is NO measured in IBMX culture (100 μg/ml) and B is NO measured in culture without IBMX.

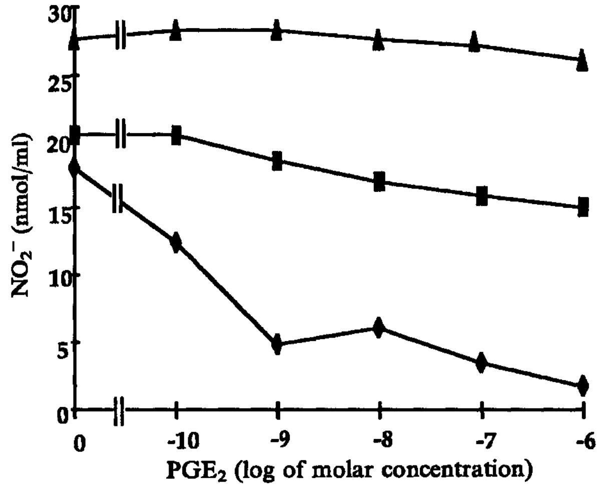

FIG. 2. Effect of $PGE_2$ in the NO produced after 3 days culture of spleen cells stimulated by LPS (1 µg/ml), IL-2 (1000 U/ml) or both. The data are expressed as the mean from one representative experiment performed in triplicate (S.E.M. < 10%). ◆, LPS; ■, IL-2; ▲, LPS + IL-2.

tion of NO was inhibited when IBMX was present in LPS-stimulated cultures (−75%), and much less for IL-2 or IL-2 + LPS stimulated cultures, −20% and −16%, respectively. LPS seems to induce cAMP accumulation since the presence of IBMX corresponded to NO inhibition, but it seems that it was not secondary to $PGE_2$ induction. Indeed, when the cultures were incubated in the presence of indomethacin, NO production was reduced similarly to that in cells cultured without indomethacin (data not shown).

The addition of IBMX produced a fall of IFN production. The IFN level (which was, respectively, 29 ± 15 and 180 ± 48 U/ml for IL-2 and LPS + IL-2 cultures) fell to 3.5 ± 0.8 and 5 ± 2 U/ml when the cultures were incubated in the presence of IBMX.

We have evaluated the effects of exogenous $PGE_2$ on NO production in spleen cell cultures with IL-2 or LPS alone, or in combination. The results of one experiment out of three are shown in Fig. 2. $PGE_2$ concentrations as low as $10^{-9}$M inhibit NO production induced by LPS alone (−73%). This effect is less important with IL-2 (−10%). $PGE_2$ does not significantly modify NO production in cultures containing IL-2 + LPS; even at a final concentration of $10^{-6}$M the decrease is only 5%.

*Effect of a PKC inhibitor on NO and IFN production:* The potential involvement of PKC in the signal transduction pathway by which LPS/IFN-γ induces NO synthesis in macrophages was suggested by Severn *et al.*[10] IL-2/receptor interaction produces a redistribution of PKC.[19] Experiments were undertaken to ascertain the role of PKC in the formation of NO and IFN as induced by stimulation of spleen cells by LPS, IL-2 or LPS + IL-2. The spleen cells were cultured in presence or absence of H-7, an inhibitor of PKC.

**Table 4.** Effect of H-7 presence on the NO produced by spleen cells cultured for 72 h with LPS, IL-2 or LPS + IL-2

| Experiment | NO variation (%)[a] | | |
|---|---|---|---|
| | LPS | IL-2 | LPS + IL-2 |
| 1 | − 65 | − 70 | − 6 |
| 2 | − 75 | − 62 | − 13 |
| 3 | − 64 | − 54 | + 9 |
| 4 | − 68 | − 62 | + 4 |
| Mean ± S.E.M. | − 68.0 ± 2.5 | − 62.0 ± 3.3 | − 1.5 ± 4.9 |

[a]% variation = A − B/B × 100 in which A is NO measured in H-7 culture (20 µM) and B is NO measured in culture without H-7.

NO production was measured after 3 days of culture and the percent variation of NO for four experiments is presented in Table 4. It was observed that H-7 induced a similar inhibition of approximately 65% for the cultures stimulated with LPS or IL-2. No inhibition was observed for cultures stimulated by LPS + IL-2. The production of IFN was inhibited by 86 ± 10% in the presence of H-7 for IL-2 cultures and 96 ± 2% for LPS + IL-2 cultures (mean of three experiments ± S.E.M.).

*Role of NO in the generation of LAK cells and IFN production:* We have found NO production by spleen cells cultured with LPS, IL-2 or IL-2 + LPS. We have examined if NO is involved in the generation of cytotoxic cells of the LAK type and in IFN production. In these experiments NO, which is derived from a terminal guanidino nitrogen from arginine, is blocked by the L-arginine analogue $N^G$-monomethyl arginine (*N*-MMA). Spleen cells were cultured for 3 days with LPS (1 µg/ml), IL-2 (1000 U/ml) or IL-2 + LPS in presence or absence of *N*-MMA (50 µM).

NO and IFN levels were measured in the supernatants and the cells were collected and tested for their cytotoxic activity against YAC cells for different effector-target cell ratios (10:1 to 0.15:1). Cytotoxic activity was expressed in lytic units. Results of four experiments are reported in Table 5.

We observed that the production of NO falls in the presence of *N*-MMA, while IFN production was always increased. There was a non-significant trend for cytotoxic activity to increase in the presence of *N*-MMA.

### Discussion

We have examined NO production in murine spleen cell cultures stimulated with either LPS, IL-

**Table 5.** The effects of *N*-MMA presence on $NO_2^-$ and IFN production and generation of cytotoxic activity in spleen cells cultured for 3 days with LPS (1 μg/ml), IL-2 (1000 U/ml) or IL-2 + LPS

| Experiment | Substance(s) added | $NO_2^-$ (nmol/ml) | | IFN (U/ml) | | Cytotoxic activity (LU)[a] | |
|---|---|---|---|---|---|---|---|
| | | Without *N*-MMA | With *N*-MMA[b] | Without *N*-MMA | With *N*-MMA | Without *N*-MMA | With *N*-MMA |
| 1 | LPS | 10 | 2 | 12 | 14 | 51 | 60 |
| | IL-2 | 13 | 5 | 18 | 29 | 500 | 667 |
| | IL-2 + LPS | 23 | 5 | 139 | 192 | 138 | 166 |
| 2 | LPS | 20 | 3 | 1 | 2 | 72 | 75 |
| | IL-2 | 20 | 3 | 11 | 83 | 625 | 1190 |
| | IL-2 + LPS | 25 | 5 | 125 | 322 | 312 | 480 |
| 3 | LPS | 11 | 1 | 3 | 5 | ND | ND |
| | IL-2 | 9 | 1 | 21 | 34 | 1049 | 1250 |
| | IL-2 + LPS | 23 | 2 | 153 | 256 | 232 | 312 |
| 4 | LPS | 15 | 1 | ND | ND | ND | ND |
| | IL-2 | 13 | 1 | 13 | 15 | 1000 | 1050 |
| | IL-2 + LPS | 23 | 1 | 100 | 1421 | 285 | 222 |

[a]LU lytic units calculated at 20% cytotoxicity/$10^7$ cells.

[b]*N*-MMA 50 μM.

ND, not done.

2 or LPS + IL-2 for 3 days. We observed similar NO production in cultures containing IL-2 and in those containing LPS. It is known that only LPS and IFN-γ are stimulating factors of NO production by macrophages.[12] The NO induced in the IL-2-stimulated spleen cell cultures was probably attributable to IFN-γ induced by IL-2.[16] The production of NO was significantly increased when LPS and IL-2 were combined in the cultures.

LPS induced low levels of IFN. The association of IL-2 with LPS induced a synergy in IFN production.[12,18] Similarly to the work of Ding *et al.*[2] in macrophage cultures, we observed that IFN-γ increases the NO induced by LPS in spleen cell cultures but different effects were observed with IFN-α/β addition. In the latter, we observed a significant reduction of NO production, whereas Ding *et al.* observed that IFN-α or IFN-β alone were not inductors of NO, but their association with LPS increased NO production although to a lesser degree than IFN-γ.

This opposing effect that we have observed, depending upon the type of IFN added to LPS, was confirmed when spleen cells were cultured in the presence of either anti-IFN-γ antibodies or anti-IFN-α/β serum. When the cultures were made in the presence of anti-IFN-γ antibodies, we observed (Table 2) a reduction of NO induced by LPS and to a lesser degree by LPS + IL-2, but there was no effect on IL-2 cultures. In the presence of anti-IFN-α/β serum a similar increase of NO was observed in LPS or LPS + IL-2 culture and a lower increase for IL-2 cultures. This result differed from that of Riches and Underwood[20] who found that the anti-IFN-α/β presence inhibited the NO release by macrophages stimulated by IFN-γ and poly IC.

Thus, IFN-α/β has immunomodulatory properties which differ from those of IFN-γ for the modulation of NO production. Different effects of these two types of IFN have been described for induction of IL-1;[21] induction of cytotoxic activity[12] and ConA and IL-2-induced proliferation of spleen T-cells.[22]

If IFN-γ is an inducer of NO, it must be emphasized that NO is an inhibitor of IFN production. Indeed when the cultures were made in presence of an inhibitor of NO, IFN production was increased.

We observe that independent of the stimulating agent, the reduction of NO by *N*-MMA was followed by a small increase in cytotoxic activity in nine of ten cytotoxic assays (Table 5). In contrast with our results, Juretic *et al.*[13] reported that reduction of NO production induced a decrease of LAK activity in rodent spleen cells. At present, we do not have an explanation for this discrepancy.

The increase of IFN production and the weak effect on cytotoxic activity in cultures made in the presence of an inhibitor of NO may be secondary to a reduction of $PGE_2$. Indeed Salvemini *et al.*[8] have shown that $PGE_2$ production was associated with NO production which activates cyclooxygenase enzymes. So we hypothesize that the fall of NO induced a reduction of $PGE_2$, which explains the increase of IFN. $PGE_2$ is known to be an inhibitor of IFN production[23] and cytotoxic activity generation.[24]

In our system LPS induced $PGE_2$ because (a) IFN production and cytotoxic activity were increased in the presence of indomethacin, which is an inhibitor of cyclooxygenase pathway;[12] and (b) IBMX which produces a cAMP accumulation inhibits NO production (Table 4). On the other hand exogenous $PGE_2$ inhibits NO induced by LPS (Fig. 2). Unexpectedly, the presence of indomethacin which should provoke an increase of NO synthesis by inhibition of $PGE_2$ production, caused a slight decrease of NO production. A similar effect was observed by Schleifer and Mansfield.[25] So cAMP accumulation in the presence of IBMX was not necessarily related to a $PGE_2$ effect. This is in agreement with the data of Piguet-Pellorce and Dy[26] who reported that the increase in histamine release by bone marrow cells stimulated by LPS plus GM–CSF, was related to an increase of cAMP, which is not related to $PGE_2$ synthesis. In contrast, $PGE_2$ can increase NO production by LPS-stimulated Kuppfer cells.[27] In IL-2 or IL-2 + LPS cultures, the presence of IBMX reduced NO production, although the inhibition was less than in LPS culture; however, IFN production was drastically reduced. On the other hand $PGE_2$ induced a slight but significant reduction of NO in IL-2 cultures, but we did not observe a $PGE_2$ effect in LPS + IL-2 cultures. This suggests that IL-2 or IL-2 + LPS induce the release of factors which counterbalance the inhibitory effect of $PGE_2$. Moreover IL-2 has been reported to partly reverse the inhibitory effect of $PGE_2$.[28]

The signal transduction pathway by which IFN-γ induces NO production remains to be established. PKC is involved in the induction of nitric oxide synthase by IFN-γ,[10] protein kinase C activity is increased by IFN-γ but not by IFN-α/β or LPS.[29] Work by Fujihara *et al.*[30] showed that PKC plays an important role in the LPS-triggered signal transduction pathway. However, other different protein kinases such as PKA via $PGE_2$ induction may be involved. There is also a link between PKA-mediated signalling and nitric oxide synthase.[11] Using H-7, an inhibitor of PKC, we observed a similar reduction of NO that is induced by LPS or IL-2. No effect was observed when the cells were cultured in the presence of IL-2 + LPS, but the presence of H-7 induced a large reduction (97%) of IFN induced by LPS + IL-2. Gessani *et al.*[31] showed that treatment of macrophages by staurosporine, an inhibitor of PKC, stimulated by IFN-γ inhibits IFN secretion.

There is a relationship between intracellular cAMP concentration and PKC activation.[32] H-7 was an inhibitor of PKC and also of PKA. One might hypothesize that the inhibition of NO induced by H-7 may correspond to the PKA inhibition of LPS, and the PKC inhibition of IL-2.

H-7 did not affect NO production in LPS + IL-2 culture but reduced IFN production. It must be emphasized that inhibition of PKC may involve the reduction of the inhibitors of NO production such as TGF-β[33,34] and/or IL-4.[35,36] On the other hand, as we have shown, it may be that IFN-α/β plays an inhibitory role in NO production, and the fall of IFN-α/β might explain the maintainance of NO levels.

We underline that two metabolic inhibitors, i.e. IBMX and H-7, which did not affect the NO level, reduced IFN production in IL-2 + LPS cultures, whereas *N*-MMA, which inhibited NO production, increased IFN levels.

### References

1. Liew FY, Cox FEG. Non specific defence mechanism: the role of nitric oxide. *Immunol Today* 1991; A17–21.
2. Ding AH, Nathan CF, Stuehr DJ. Release of reactive nitrogen intermediates and reactive oxygen intermediates from mouse peritoneal macrophages. Comparison of activating cytokines and evidence for independent production. *J Immunol* 1988; **141:** 2407–2412.
3. Weisz A, Oguchi S, Cicatiello L, Esumi H. Dual mechanism for the control of inducible-type NO synthase gene expression in macrophages during activation by interferon-γ and bacterial lipopolysaccharide:transcriptional and post-transcriptional regulation. *J Biol Chem* 1994; **269:** 8324–8333.
4. Kurland JL, Bockman R. Prostaglandin E production by human blood monocytes and mouse peritoneal macrophages. *J Immunol* 1978; **147:** 952–960.
5. Hamilton TA, Gainey PV, Adams DO. Maleylated-BSA suppresses IFN-γ mediated Ia expression in murine peritoneal macrophages. *J Immunol* 1987; **138:** 4063–4068.
6. Bulut V, Severn A, Liew FY. Nitric oxide production by murine macrophages is inhibited by prolonged elevation of cyclic AMP. *Biochem Biophys Res Commun* 1993; **195:** 1134–1138.
7. Raddassi K, Petit JF, Lemaire G. LPS-induced activation of primed murine peritoneal macrophages is modulated by prostaglandins and cyclic nucleotides. *Cell Immunol* 1994; **149:** 50–64.
8. Salvemini D, Misko P, Masferrer JL, Seibert K, Currie MG, Needleman P. Nitric oxide activates cyclooxygenase enzymes. *Proc Natl Acad Sci USA* 1993; **90:** 7240–7244.
9. Bakouche O, Moreau JL, Lachman LB. Secretion of IL-1: role of protein kinase C. *J Immunol* 1992; **148:** 84–91.
10. Severn A, Wakelam MJO, Liew Y. The role of protein kinase C in the induction of nitric oxide synthesis by murine macrophages. *Biochem Biophys Res Commun* 1992; **188:** 997–1002.
11. Brune B, Lapetina EG. Phosphorylation of nitric synthase by protein kinase A. *Biochem Biophys Res Commun* 1991; **181:** 921–926.
12. Vaillier D, Daculsi R, Gualde N. Effects of lipopolysaccharide on interleukin-2-induced cytotoxic activity of murine splenocytes cultures: role of prostaglandin $E_2$ and interferons. *Cancer Immunol Immunother* 1992; **35:** 395–400.
13. Juretic A, Spagnoli C, von Bremen K, *et al.* Generation of lymphokine-activated killer activity in rodents but not in humans is nitric oxide dependent. *Cell Immunol* 1994; **157:** 462–477.
14. Green C, Wagner DA, Glogowski J, Skipper PL, Whisnok JS, Tannenbaum SR. Analysis of nitrate, nitrite and 15n-nitrate in biological fluids. *Ann Biochem* 1982; **126:** 131–138.
15. Hudson L, Hay FC. *Interferon in Practical Immunology.* Oxford: Blackwell Scientific Publications, 1980; 296–298.
16. Torres A, Farrar L, Johnson HM. Interleukin 2 regulates immune interferon (IFN-γ) production by normal and suppressor cell cultures. *J Immunol* 1982; **128:** 2217–2219.
17. Le J, Lin X, Henricksede Stefano D, Vilcek J. Bacterial lipopolysaccharide-induced interferon production. Role of interleukin-1 and interleukin-2. *J Immunol* 1986; **136:** 4525–4530.
18. Blanchard K, Djeu JY, Klein TW, Friedman H, Steward E. Interferon-γ induction by lipopolysaccharide: dependence of IL-2 and macrophages. *J Immunol* 1986; **136:** 963–970.
19. Farrar WL, Anderson WB. Interleukin-2 stimulates association of protein kinase C with plasma membrane. *Nature* 1985; **315:** 233–235.
20. Riches DWH, Underwood GA. Expression of interferon-β during the triggering phase of macrophage cytocidal activation. *J Biol Chem* 1991; **266:** 24785–24792.
21. Newton RC. Effect of interferon of human monocyte secretion of interleukin-1 activity. *Immunology* 1985; **56:** 441–449.
22. Klimpel GR, Infante AJ, Patterson J, Hess CB, Asuncion M. Virus-induced interferon α/β (IFN-α/β) production by T cells and Th1 and Th2 helper T cell clones: a study of the immunoregulatory actions of IFN-γ *versus* IFN-α/β on functions of different T cell populations. *Cell Immunol* 1990; **128:** 603–618.
23. Betz M, Fox BS. Prostaglandin $E_2$ inhibits production of Th1 lymphokines but not of Th2 lymphokines. *J Immunol* 1991; **146:** 108–113.

24. Longley RE, Stewart D, Roe KG, Good RA. Inhibition of murine lymphokine-activated killer (LAK) cell activity by adherent cells. *Cell Immunol* 1989; **121:** 225–232.
25. Schleifer KW, Mansfield JM. Suppressor macrophages in African trypanosomiasis inhibit T cell proliferative responses by nitric oxide and prostaglandins. *J Immunol* 1993; **151:** 5492–5503.
26. Piquet-Pellorce C, Dy M. Effect of lipopolysaccharides on histamine synthesis by hematopoietic cells. *Cell Immunol* 1991; **135:** 360–371.
27. Gaillard T, Muslch A, Busse R, Klein H, Decker K. Regulation of nitric oxide production by stimulated rat Kupffer cells. *Pathol Biol* 1991; **59:** 280–283.
28. Walker C, Kristensen F, Bettens F, Deweck AL. Lymphokine regulation of activated (G1) lymphocytes. I. Prostaglandin E2-induced inhibition of interleukin 2 production. *J Immunol* 1983; **130:** 1770–1773.
29. Hamilton TA, Becton DL, Somers SD, Gray PW, Adams DO. Interferon-γ modulates protein kinase C activity in murine peritoneal macrophages. *J Biol Chem* 1985; **260:** 1378–1381.
30. Fujihara M, Connoly N, Ito N, Suzuki T. Properties of protein kinase C isoform (βII, ε, and ζ) in a macrophage cell line (1774) and their roles in LPS-induced nitric oxide production. *J Immunol* 1994; **152:** 1899–1906.
31. Gessani S, Bellardelli F, Pecorelli AA, Puddu P, Baglioni C. Bacterial lipopolysaccharide and gamma interferon induce transcription of beta interferon mRNA and interferon secretion in murine macrophages. *J Virol* 1989; **63:** 2785–2789.
32. Friedrich B, Cantrell DA, Gulberg M. Increased cyclic cAMP levels block interleukin 2 induced protein kinase C substrate phosphorylation but not the mitogenic response. *Eur J Immunol* 1989; **19:** 1111–1116.
33. Ding A, Nathan F, Graycar J, Derinck R, Stuehr D, Srimal SA. Macrophage desactivating factor and transforming growth factors-$\beta_1$, -$\beta_2$ and -$\beta_3$ inhibit induction of macrophage nitrogen oxide synthesis by IFN-γ. *J Immunol* 1990; **145:** 940–944.
34. Vodovotz Y, Bodgan C, Paik J, Xie QW and Nathan C. Mechanisms of suppression of macrophage nitric oxide release by transforming growth factor β. *J Exp Med* 1993; **178:** 605–611.
35. Suk K, Somers SD, Erickson KL. Regulation of murine macrophage function by IL-4: IL-4 and IFN-γ differentially regulate macrophage tumoricidal activation. *Immunology* 1993; **80:** 617–624.
36. Bodgan C, Vovovotz Y, Paik J, Xie QW, Nathan C. Mechanism of suppression of nitric oxide synthase expression by interleukin-4 in primary mouse macrophages. *J Leuk Biol* 1994; **55:** 227–232.

ACKNOWLEDGEMENTS. This work was supported by grants from Ligue Nationale Contre le Cancer. The authors acknowledge Ms M. Birac for her secretarial assistance.

**Received 24 October 1995; accepted in revised form 8 December 1995**
